# Attachment-Based Family Therapy for Adolescent Substance Use: A Move to the Level of Systems

**DOI:** 10.3389/fpsyt.2019.00948

**Published:** 2020-02-05

**Authors:** Andrew J. Lewis

**Affiliations:** Discipline of Psychology, Murdoch University, Perth, WA, Australia

**Keywords:** adolescents, substance abuse, attachment, systems theory, family-based intervention

## Abstract

This paper provides an account of the theoretical basis of a family-based intervention called Behaviour Exchange and Systems Therapy (BEST). The model described here has also been applied to adolescents with substance abuse and other mental health problems such as depression and anxiety disorders in both children and adolescents. Evaluative studies of the model have been published including randomised clinical trials as well as qualitative analyses. The current paper discusses a theory of the family system as a discourse and represents an integration of aspects of attachment, psychoanalytic, and systems theories. Key concepts elaborated are the attachment-family system, the family as a single discourse, the use of segregation as a defense in relation to trauma and loss and its manifestation in a family narrative, and the role of the family secure base in affect regulation. The paper also briefly describes specific treatment techniques that are derived from the theoretical model. Our approach has wide application as a discourse focused treatment for children and adolescents using a family systems approach. Future work requires the comparison of this model to similar attachment-based models of intervention for children and families, further development and validation of measures able to be used for whole families in a clinical setting, and further empirical demonstration of treatment efficacy in a variety of clinical settings.

John Bowlby opened his 1948 paper *The Study and Reduction of Group Tensions in the Family* by writing: “Child guidance workers all over the world have come to recognise more and more clearly that the overt problem which is brought to the clinic in the person of the child is not the real problem; the problem as a rule we need to solve is the tension between all the different members of the family” (Bowlby, 1948, p.123). The clinical approach we describe in this paper is something of a return to the systemic emphasis we find in this comment. Such a return to the systems level of the family can be distinguished from the internalized cognitive model of attachment based on a representational and therefore individuated model of attachment theory. When attachment theory is thought of as a discursive-relational model, it fits neatly with both interpersonal and systemic clinical approaches. As we can hear in the comment above, from the outset, Bowlby clearly emphasized the *child and family* in his clinical thinking (1).

The current paper is focused on the elaboration of the key principles of such a discursive-relational approach, and a description of the treatment techniques of *Behaviour Exchange and Systems Therapy* (BEST). Our research team based in Melbourne and Perth have been developing the theory and practice of BEST in different forms for two decades. These interventions are family-based interventions and can be delivered either to individual families or with small groups of families. Initially, these interventions focused on the parents of adolescents presenting with substance abuse, but the model evolved over time into a whole of family approach and was adapted to also serve as a treatment for adolescent depression and anxiety (2, 3). Our work now is extending the approach to interventions for children under 12 years of age.

Clinical trials our team have being running in Australia have accumulated good evidence to show that the approach can effectively treat a range of adolescent mental disorders. The studies show that improvement in adolescent mental health is typically accompanied by improvements in family functioning and notably improvements in the parent’s mental health (2–8). Previously our research group has also published qualitative studies of participant experiences as well as a description of the main features of the program for the treatment of adolescent depression called BEST-Mood (5, 9, 10). These make up a rich quantitative and qualitative dataset which forms the background to this theoretical paper.

## Discourse, Narrative, and Dialogue

There is an increasing use of the term “attachment-based therapy” referring to relational approaches and these are generally considered to have the broad goal of promoting attachment security between parents and children (11). An attachment-based approach minimally adopts a dyadic view of inter-subjective communication (12) rather than treating an individual. Such models of therapy have their origins in the clinical approach originally described by Bowlby (1), but the clinical application of attachment theory has been elaborated by many others, usually, but not exclusively, in relation to a broadly psychodynamic framework (13–18). Alongside the work of other groups focused on adolescent mental health, we are interested in how attachment patterns are perpetuated within a family system and how such an understanding can inform interventions (19, 20).

Our clinical interests in an integration of attachment theory and family systems has led us to propose a conceptual shift from a representational model to a discourse model. The most common way of clinically interpreting attachment is derived from the “working model” concept. This is thought to be an individuated representational-cognitive model (21) and it extends the cybernetic notions of signalling in Bowlby’s evolutionary-development framework. The concept of an “internal working model” originates in the attempt to explain how early relationship experiences are carried forward as enduring styles of interpersonal relations and modes of regulating affects. Attachment theory’s next major development occurred with Mary Main’s work on the manifestation of attachment patterns within adult narratives and in developing this theory it is of importance to recall that she was drawing very directly on H.P. Grice’s categories of conversational coherence (22, 23). Attachment classifications based on the coding of the Adult Attachment Interview (AAI) were able to reliably identify very specific discursive features of language use. For example, this includes the mode of recall of early attachment memories, narrative accounts of separation, loss or challenging interpersonal experiences, the subject’s capacity to mentalise about aspects of their parent’s relationship. Overall patterns of autonomous, dismissive and preoccupied conversational styles emerge across the full interview. These components are rated in terms of an overall *coherence* of discourse, reflecting the integration, and consistency of the narrative.

This shift to the level of representation has given rise to a range of discourse-based measures of attachment and generated a substantial body of evidence to validate the concept of attachment discourse. Discourse based assessments of adult attachment have been more recently developed to analyse responses to images (Adult Attachment Projective- AAP) (24), a secure-base script method (25), and similar ideas have been applied to the analysis of child play narratives in response to structured attachment stimuli (26). This discourse model in particular is fundamental to many clinical applications of attachment theory, and certainly attracted a renewed exchange with psychoanalytic theory in the 1990s (27–29). Empirically, a number of important studies have now shown relationships between attachment discourse measures and broader aspects of family discourse. For example, studies found that mother’s scripts of secure narratives were related to both the child’s degree of attachment security, and the mother’s narrative style and emotional language when reminiscing about shared experiences (30). The researchers suggested that their findings should be understood in terms of the way mother-child dyads discuss emotion-laden content. Similar findings have been reported in high risk samples with histories of child maltreatment (31)

Now, in some clinical models, attachment theory has been applied to a family by supposing that each family member interacts with the other members on the basis of their internalised model of prior relationships. In effect, this view sees family interactions as reflecting individual attachment histories preserved as a generalised “Attachment State of Mind”. However, by shifting this framework to the level of a systemic approach, a family therapy can more effectively focus on the family as a single discursive system. To elaborate this idea, we can say a single-family discourse, viewed synchronically, consists of the set of statements in a given family. However, the term “discourse” does not simply refer to an individual’s speech acts, but its reception within a given social context. In this sense, discourse requires dialogue. In our therapeutic application, the social context is considered to be the family and the dialogue includes not only speech, but also any actions which have a communicative effect. Such styles of interacting constitute the family discourse which we suggest has consequences for the formation and perpetuation of attachment relationships.

From a diachronic perspective, the family discourse has an historical legacy in the discourses of the parent’s own family of origin. Such histories are subjected to a continual process of integration over time into the current family discourse. For example, once a parental couple is formed there is a major integration of two family histories. Similarly, when children are born, there is a further elaboration of the discourse in terms of the experiences of parenting each of their offspring. At any given moment, the family discourse *constructs* a position and role for each family member. Each family discourse consists of an implicit set of rules for what can and cannot be said, and what can and cannot be done (32). This is quite a different perspective to seeing a family as a conglomerate of “internal working models”.

The difference between family discourse and internal working models has a number of consequences. First of all, a discourse is not an internalizes representation of a relationship, it is an external articulation or set of communicative actions. The family’s discourse is derived from historical experiences and material which is intergenerational, but as a synchronic function, it is always updating itself and seeking to retrospectively make sense of the past. The discourse is also able to adjust to new circumstances in the present. The family narrative is the process whereby a family draws upon the resources available within its current discourse to construct a temporal account of its history. So, discourse and narrative are closely related, but distinct concepts. The family discourse at any given time is a major work of integration and an attempt to reach a degree of coherence through a process of dialogue, but coherence is only an ideal or a goal. The family discourse is analogous to a myth and could be described in terms of Levi-Strauss’s celebrated concept of *bricolage*, since it is pieced together from various threads of narrative, a reconstructive and a retrospective process in which there are always revisions and contested attempts to renegotiate the meaning and significance of the past (33). There is no possibility of testing the correspondence of the narrative account with the actual historical events in the therapeutic setting. There is only the degree of coherence and consistency of statements within the discourse.

On this basis, we conceptualise our treatment goal as firstly to improve the degree of organization and discursive coherence of the attachment-family system. Any family discourse is on a continuum of being more or less coherent at any given time. The clinical goal is a pragmatic one: for the family’s discourse to be coherent enough to provide a platform for family life. Second, the approach is based on the assumption that targeted and strategic interventions designed to promote changes in the relationship between parents and children can modulate both communicative actions and affective states for both parents and children (34). Changes in ways of speaking, modes of interacting, and different ways of experiencing affects lead to overall shifts in the functional operation of the family. This entails identifying impasses where the dialogical process has broken down or “frozen”. We conceptualize therapeutic changes as shifts in the family discourse. There are two major ways in which the dialogue breaks down— both of which fall outside discourse as such. These are the experience of unresolved trauma or loss, and second the enactment of uncontained affect.

## The Limits of Discourse: Trauma, Loss, and Enactment

A major theme in our clinical work is the predominance of experiences of loss and trauma when undertaking our clinical work with families. There are painful memories, attempts to represent raw events, traumas, loss, bereavement, illnesses— and these may constitute gaps and elisions, discursive ‘black holes’ in the realm of what is unspeakable. We find there is particularly rich material in attachment theory to draw on here, especially research on disorganized/unresolved attachment in both the behavior of infants, but particularly the attachment discourse of unresolved adults. Bowlby’s work makes a major contribution to the psychology of loss and trauma by showing how permanent losses, prolonged separation from the primary attachment figure, experiences of abuse and neglect are experienced as major assaults on the coherence and function of the attachment system (35). Later research on adult attachment revealed that the transmission to infants of unresolved experiences of loss and trauma can be predicted even from the attachment discourse of pregnant women (36). This implies that the origins of an offspring’s disorganized attachment are somehow present in the mother’s attachment related discourse, prior to even interacting with their infant (37, 38). Therapeutically, the fundamental question here is how to intervene to prevent or reverse such transmission. This is one of the core questions of any attachment-based therapy.

Explanations of this transmission of experiences of trauma and loss across generations generally refer to Main and Solomon’s characterization of disorganized infants. These authors employed the ethological concept of “conflict behavior” to explain the paradoxical dyadic interactions of disorganized mother-infant dyads (39). Others have pointed out the similarity between this concept and the systems theory concept of the double-bind (40). Bateson referred to the double bind as “some sort of tangle in the rules” or a confusion between the object language and the metalanguage such that several contradictory statements simultaneously direct a behaviour (41, 42). The disorganized-disoriented infant provides a good example of a double bind: the infant is motivated to respond to a threat by seeking the protection and proximity of their primary attachment figure, but in doing so, they encounter not comfort and assuagement, but threat, fear, helplessness, alarm, panic, aggression, and so on— their attachment system is frozen by an unresolvable paradox due to self-contradictory statements. The point made by attachment theories is that the impasse in the infant’s behavior is both precipitated and maintained by the contradictory interactions and communications of the attachment figure.

Mary Main’s 1991 paper provides a cognitive explanation by introducing the idea that disorganized discourse results from lapses in the metacognitive monitoring of conversational rationality. She distinguished between single versus multiple models of attachment (43) referring to the cognitive underpinnings which allow multiple and contradictory models of the same aspect of reality. In effect, Main is using the same kind of explanation as Bateson: a confusion of object language and meta-language. The metacognitive monitoring of the coherence of discourse fails at the point where it needs to provide a consistent and coherent account of trauma or losses.

We generalize this idea to the family discourse and note that contradictory or segregated accounts of a given traumatic experience are often encountered in the clinical setting. Mary Main notes the vivid examples of segregated models of attachment given in Bowlby’s discussion of parent’s denial and distortion of traumatic events which a child has directly observed: a child may have witnessed a parent’s suicide, only to be told that he had died of an illness or accident (43). Bowlby also referred to examples of a child who found her father’s body hanging in a closet only to be told he had died in a car accident (35). Much of this has been articulated in similar terms within psychoanalytic theory, but our application to work with families is to add the suggestion that the split is not simply internal to the ego, and we do not conceptualise it as an “intrapsychic defense” but think of these contradictions as frozen elements in the family discourse.

The failure to integrate such experiences into a family discourse impacts the family’s mode of communication and interaction. Instead of being integrated into the narrative process, sometimes these experiences repeat as triggered enactments and incongruous displays of affect. Enactment can be thought of as a pre-representational means of processing affect through a non-communicative action. Our view on the relationship between discourse, which is by definition social, and affect, which is individually embodied, is related to our concept of enactment. Enactment as a concept has its origins in the psychoanalytic tradition where it is related to repetition compulsion (44). A great deal more would need to be said about the relationships between attachment models of affect and the psychoanalytic drive theory, but that is well beyond the scope of this paper. The key point clinically is that the management of contradictory family discourses is closely related to conflicted and threat activated emotional systems. The escalation in parent-child conflict is well known in the literature as a very strong predictor of adolescent mental disorder (45). Families often present with narratives of contests for domination, patterns of threat and counter-threat, adolescents testing their power in response to threat, or using withdraw. Adolescence brings new modes of enactment such as threats to leave home, self-harm, suicide attempts, taking drugs, and so on. Such acts typically occur in the absence of family dialogue and proximity seeking. Addressing enactment, promotion of dialogue and resolving contradiction, defusing patterns of threat and counter-treat, are therefore crucial concepts in the clinical model.

## Review of Attachment Related Predictors of Addiction

Before elaborating these ideas, it is valuable to very briefly review the evidence that can be used to justify a focus on the whole family in relation to adolescent substance abuse. This requires looking broadly across several areas of research in order to understand the kind of experiences and histories which should be the focus of family interventions where adolescent substance abuse is a salient feature. It is important that any psychological theory be posed in terms that are consistent with the most current neurobiological findings of the corresponding phenomena. A number of researchers have pointed out the parallel between psychological processes related to attachment figures, both parental and romantic, and similar mental dispositions in states of addiction (46, 47). Papers are now emerging integrating neurobiological and psychodynamic perspectives into a developmental model on the basis of the findings linking attachment and addiction (48). One neurobiological model of addiction suggests that deficits in a person’s ability to derive rewards from sustained interpersonal or intimate relationships impels reward seeking through the repeated use of psychoactive substances which stimulate these same dopaminergic brain regions (49). There are animal studies in which exposure to early life stressors predispose to vulnerability to later substance use which point to neural mechanisms involving alteration of neural reward pathways and separation distress regulation (50). Another line of animal research has proposed gender specific pathways beginning in adolescence. Females predisposed to a heightened stress response are more liable to seek substances as a means of ameliorating high stress reactivity. Males are more likely to respond to chronic stressors with a blunted stress reactivity and their attraction is to substances which increase arousal, increase social capacity, or provide novel sensation such as cocaine and methamphetamine which block dopamine reuptake, and increase dopaminergic activity (51, 52).

The psychological and developmental literature already contains several excellent reviews that have examined the empirical findings showing the relationship between a variety of measures of attachment and different kinds of addiction (53, 54). While it is well accepted that addiction results in the deterioration of the quality of close relationships, Fairbairn’s review showed that longitudinal studies have established that attachment insecurity prospectively predicts the development of later substance problems irrespective of the type of measure used. Another interesting finding to come from this review was that the relationship between insecure attachments and substance use was less pronounced in older age groups. The same pattern has been observed in other reviews on the wider relationship between attachment and psychopathology (55) pointing to the particular importance of the interaction of attachment and developmental processes in adolescence. There have also been interesting findings suggesting that different types of insecure attachment may influence preferences for different substances of abuse (56).

Unfortunately, at his point in time, the current evidence includes only a handful of studies examining the attachment related discourse of substance using adolescents *via* their performance in the AAI or AAP. These include findings of a strong association between preoccupied-enmeshed and substance use in a sample of orphans (57). The other adolescent studies of this type have found associations between avoidant-dismissing and unresolved-disorganized representations in a variety of different substance using groups (54). Adult studies of substance abuse have found associations with Lyons Ruth’s hostile-helpless pattern and also with the Main coding of unresolved/disorganized (58). The main findings of discourse-based measures in adolescence suggest associations between substance use and dismissing forms of insecurity and reasonably consistent findings of high rates of unresolved/disorganized attachments.

The place of trauma and loss in the clinical treatment of patients with substance abuse is also well documented in other studies. It is well established that both Posttraumatic Stress Disorder and bereavement predict increases in substance use and the development of substance use disorders (59, 60). Such findings are consistent with studies on relationship qualities within families showing that adolescent substance abuse is predicted by factors such as low family cohesion, family member enmeshment, and a parenting style known as affectionless control (61, 62). Such findings provide evidence to support the relevance of treatment and prevention goals designed to improve a person’s capacity to form and preserve close relationships, be those within a family context or in other close relationships, as a means of either prevention or treatment of substance abuse (63). With these factors in mind we can now elaborate five therapeutic strategies that have been developed in our clinical work.

### The Adolescent as Proxy: The Referral and Presenting Problem

A first area to comment on is the referral process where adopting a systems approach has substantial advantages over the individual model typically used in adolescent mental health services. There often are major challenges in engaging adolescents in any form of psychological treatment and, at the time of initial referral by parents or professionals, the adolescents themselves are sometimes not willing to present for treatment. Within our model the sessions can commence with whichever members of the family are willing to attend. An adolescent’s refusal to attend sessions can become a powerful position in the system and can be thought about clinically as a form of communicative action. Refusal may be a signal of a wider refusal to be part of the family’s everyday life. This is because underlying the referral of the adolescent and the presenting problems of “substance abuse” is a clinical encounter with a family who often are at a point of fragmentation. At the point of referral, the typical situation is one of breakdown in the major attachment relationships across the family. This is consistent with the empirical findings of a bidirectional relationship between attachment insecurity as both an antecedent predictor of substance use disorder, but also that substance use induces further deterioration in the quality and functioning of close relationships (54). In some cases, there is strong intergenerational transmission and one is dealing with the adolescent offspring of parents with a history of substance abuse (64). In the context of the treatment of adolescents still residing in their family of origin, clinical referral often comes at the end of this vicious cycle of deteriorating relationships generating a point of crisis in the family-attachment system. There are important conceptual and clinical questions to be considered even at the point of referral. Who is actually making the referral for treatment? Who in the family system is most willing to consider change? What impasse within the family does the adolescent represent? Referral is therefore not considered to be the referral of an individual with a “mental disorder” requiring that individual to attend and receive treatment. Instead we consider referral to be the referral of a family, as a system, at a point of crisis in that family’s history.

### A Letter of Invitation: From Helplessness to Action

One of the most common comments from a parent at the commencement of the treatment is “It feels like there is *nothing* I can do.” (5). Our clinical work suggests that at the commencement of sessions parents have often adopted a helpless position and probably for quite a long time prior. The concept of helplessness (*Hilflosigkeit*) has deep roots in the psychoanalytic tradition and was revived as an attachment concept by Lyons-Ruth (65). In the parent’s helplessness, one can also recognize a specific dynamic, common in child and family therapy, in which the more helpless the parent, the more domineering the child. It is a family situation of great isolation and disconnection. From a relational perspective, we can see that substance abuse acts as a freezing point in the family discourse and its dialogical movement. On the one hand the adolescent is focused on addictions and these are a one-sided affair, that is substances, while generally reliable, do not “relate back” or make relational demands (66). Addiction for an adolescent belies a breakdown in the trust that another is capable or willing to respond to their interpersonal and relational needs. On the other hand, the parental helplessness and withdrawal is the parental counterpart and complicit with this freezing in the family system’s dialogue.

The first response to this sense of helplessness and isolation is to discuss with parents, either alone or in a small group, the many small ways that they can be effective in relation to their adolescent’s problem, how change is incremental and requires persistence, and how they can take action to contribute to improvement in family life. It is critical to do this in a positive way which is very distinct from implying that parents are somehow responsible for their adolescent’s disorder. One approach that has been used with some success is to ask parents to write a letter of invitation to their adolescent, telling them that they are attending a group, the concerns they have about current family life, and expressing a desire for change and inviting the adolescent to join with them in attending sessions. The parents work on this letter over the initial sessions of treatment, consulting with the therapists and sharing drafts for comment. Often the parents will be lacking in confidence to produce the letter, feel it will be a useless gesture, or use the writing as a vehicle to vent their own anger and frustration. All this is worked through. The adolescent often receives the letter with surprise and it generates some curiosity. It is both a challenge to the state of helplessness and serves as a gesture of sending a message indicating that the parents are taking the initiative, stepping up as open to dialogue and agents of change.

## The Oxygen Mask: Rebuilding the Family Secure Base

In many cases, what Bowlby referred to as the “emotional atmosphere” of the family is characterised by a vicious cycle of uncontained affect and its behavioral enactment (67). In other literature, a similar idea might be presented under the concept of expressed emotion. There are common parent-child dynamics in which adolescent withdrawal or aggression triggers parental distress and helplessness. It is clear that the situation with the adolescent is activating basic affective systems in the parent including panic, catastrophic or escalating fear, despair, and anger/aggression (68). As mentioned above, the attachment perspective understands these affective systems as threat activated affects which trigger basic survival systems. They do so by shutting down affiliative and care-giving motivational systems. We also find that systemically these vicious cycles of affect and enactment can escalate to such a degree that they precipitate a premature rupture in the family-attachment system. The adolescent seeks to achieve a kind of pseudo-independence in which they sometimes leave or sometimes remain physically within the family, but are psychologically cut off within the family, unable to access any sense of security *via* intersubjective relations within the family. This may take the form of an externalising presentation in the context of substance use which often consists of various conduct and “anti-social” problems, taking up with their peer group, in some cases spending little or no time in the family unit. Another permutation is the withdrawal of a depressed adolescent within the family— the parents describe them as moody, difficult to reach or living in a virtual world of social media (10).

An important concept derived from attachment thinking which we use to both understand and respond to such situations is John Byng-Hall’s concept of the “secure family base”. He uses this term to describe the family foundation from which an adolescent can safely explore their social world (11, 69). The notion of a secure family base refers to the parental function and it assumes to some degree a unified parental position. We have encountered several obstacles to the parents facilitating the family operating as a secure base.

First, we often encounter a challenge within the parental couple itself who, under enormous stress, find it difficult to present a unified front. Instead it is common that they turn on each other and split off into polarised reactions to a challenging situation. This is understandable within a context where each parent brings their own attachment history, styles of defense, and their own ways of having traversed adolescence and the position of parenthood. Therapeutic discussion of these three moments: the parent’s own childhood attachment histories, their traversal of adolescence, and their assumption of parenthood— can be a source of significant therapeutic gain. The therapist needs to be looking out for when this parent has been able to make use of a reparative attachment experience with a reflective other. For example, it is not unusual that a parent may have worked-through an adolescent period of rupture with their own parents, but later made reparation when they formed a new couple relationship by making use of new capacities derived from their romantic relationship.

Second, we frequently encounter within disorganized family dynamics histories of role reversal emerging over early and middle childhood. The same dynamic has been described using various different terms in psychoanalytic, systems, and attachment theory (70, 71). Role confusion and reversal begins with the primary attachment figure not providing care to the infant, but in numerous different ways and circumstances seeking or requiring that care themselves. As noted by Lyons-Ruth, it is not unusual to also uncover in such parents histories of victim/aggressor relational patterns, patterns of withdrawal in the face of the child’s attachment demands, and a critical failure to regulate the child’s attachment need in those moments where assuagement of distress is most needed (72). The child’s defensive positioning within this dynamic as “parentified” takes up a subjective position of control in their relation with others, an objectification of others as objects to be controlled, and perceives that there is an absence of any anyone else “taking control”. There are elements of both grandiosity and narcissism at play in the child’s position and in adulthood this can develop into a personality style which seems to exude a high degree of “competence”. However, from a clinical point of view, the predominance of role reversals between caregiver and care-receiver bellies a major alteration in family structures by placing the child in the dominant and controlling position. The most obvious form this takes in adolescence is a control that takes an aggressive and commanding form, but equally the adolescent’s withdraw in the more internalizing presentations can be seen as a mode of control.

The therapeutic response is twofold. First, to rebuild the family as a secure base and this entails numerous different techniques designed to allow parents to contain their distress, redefine their roles as supportive, see themselves as setting examples of coping with stressors and taking responsibility for problems. There are also a range of techniques to rejuvenate the dialogue by parents showing they are willing to change and adapt, communicate, compromise, and negotiate and to expect the same of their adolescents. Here amongst other approaches, we make use of a metaphor based on the use of the Oxygen mask— “In the aeroplane, safety instructions suggest that the parent secure their oxygen mask *before* assisting their children…”

The idea of this and various other components of the treatment is to promote the parent’s adoption of the position of the family secure base. This foundational position could also be likened to a sounding board, which facilitates the reconstruction of family discourse. The second aspect presupposes the first has been achieved to some degree and is based on an encouragement that in the context where the family has achieved a secure base, an adolescent will seek to explore. We reframe such “explorations” in terms of their importance in the adolescent resuming their developmental pathway towards autonomy, to re-negotiating their relationship with their parents as they enter adulthood, and are able to make use of the availability and receptivity of their parents in new ways.

## Red Buttons: Intersubjective Regulation of Affect

There are important systemic factors which perpetuate family conflict. It is also apparent that conflict based on mutual aggression can become a vicious cycle of affective dysregulation, where aggression triggers the escalation of threat which in turn triggers further aggression, described by Bateson using the concept of schismogenesis (73). This cycle can become particularly vicious when, as occurs for many parents in our intervention, the idea of the adolescent’s separateness generates panic. Separateness is not greeted as a developmental achievement, but as a threat to their child and the integrity of the family. Parents then see it as their role to intervene to “prevent damage” occurring, and this can be very acute in relation to drug use, but this implicitly sends a message to their adolescent that they are considered “incompetent” or cannot “cope on their own”. It also sends a message that their adolescent is in grave danger, but the adolescent is considered to lack the skills to keep themselves safe. In our experience, this can generate an emotional atmosphere which does have features of alexithymia, but with a heightened sense of panic and an aggressive battle for control. This drives the adolescent further away and undermines the adolescent’s developmental process of autonomy seeking, building social confidence, and sometimes taking risks.

These are themes discussed in our intervention around a series of metaphors. These take the form of stories of separation, autonomy, risk, and adventure designed to evoke discussions of separation as a key developmental process in adolescence. The generation of the family as a secure base requires that the parents stabilise their affective responses to these sometimes-threatening themes. Such stabilization occurs through discursive coherence and the capacity to speak about and think about, rather than enact these powerful affective experiences.

Clinically addressing family conflict and aggression is a core part of our approach. Above all we emphasize that effective communications cannot occur in the context of conflict and hostility. The first step is often to help families recognise the degree of aggression and conflict inherent in many of their interactions. It is critical to have therapeutic discussions naming the kinds of emotional experiences the parents are having. We also discuss common “hot spots” which are points in family life which tend to generate conflict- getting out of bed, going to bed, getting to school on time, etc. We often appeal to the idea that parents need to model taking control not of their child, but of their own emotions, to recognise when they are feeling “out of control” and to curtail interactions based on that recognition. Parents are encouraged to regather their self-control and then return to seek dialogue. Often this is discussed, modelled and even roll played with the therapists.

Sometimes, a “circuit breaker” is needed by way of intervention. One idea arose from recounting the experience of one of the participants. One of our dads used to talk a lot in the sessions about his “Red buttons”— these were the ones his daughter knew well how to push! And the two of them were often triggering aggression in each other to the point where they were often unable to inhabit the same space. One day, anticipating an argument, the dad came into his daughter’s room with an actual red button stuck on his sleeve and said to her “Do you just want to push it and get that bit over with and then we can discuss the issue” She laughed and there was a shift … This has become a story we tell within sessions since it very nicely illustrates how a parent can redirect what was typically an aggressive enactment onto a discursive level through the use of humour.

## Bumps in the Road: Integrating the Narrative of Loss and Trauma

Therapeutically, there is great benefit in addressing segregated systems at a family level. There are a variety of techniques that encourage a family unit to collectively work through their narrative of traumatic experiences, or family losses or other major setbacks. The approach is to ensure that this is done in a manner in which all family members can contribute and where therapists are proactive in seeking clarity, in asking for other’s versions of events, and to encourage a goal of “setting the record straight”. In our model, we used a simple drawing technique called “bumps in the road” in which family units are asked to draw their family road trip along a “rocky road” with the bumps and pitfall labeled along the way. Who is driving their car, who are the passengers? When has it needed repairs? It generally takes some time before the family is ready for this task following earlier work to build the family secure base and a sense of trust in the therapeutic process. Often there are highly impactful sessions where a sense of both clarity and the theme of “how did we survive it all” emerges.

## Conclusions

To conclude, this paper has tried to give a sense of how attachment research and theory can be used to inform and develop a family-based treatment approach for adolescents with mental health issues— including substance use. There is compelling evidence that there are higher rates of attachment insecurity in substance using adolescents and also strong evidence for histories of trauma, loss, and family conflict. Alongside what we now know of the neurological processes involved in addiction and their links to social affiliative systems, this justifies the need for an attachment approach to such family-based treatments.

The basic theoretical commitment of the BEST approach is based on the claim that the “move to the level of representation” in attachment theory, can be reconsidered as a properly inter-subjective and linguistic model, compatible with family systems theory. This is broadly consistent with those approaches which could be called the “linguistic turn” in psychotherapy. These approaches all emphasize language as a means to generate meaning in shared patterns of communication, and that meaning can take the forms of action and interaction. There are limits to meaning generation in terms of enactment and overwhelming experiences of affect. The concern with language, meaning, dialogue, and narrative are widely shared by systemic approaches such as narrative therapy (74) postmodern therapy (75) and dialogic family therapy (76), and by contemporary psychodynamic approaches such as Lacanian and Neo-Kleinian psychoanalysis. This paper has attempted to bring these elements of systems and psychoanalytic thinking together with discourse-oriented research within attachment theory (40, 77, 78).

Certainly, BEST is not the only family systems model to draw on attachment theory and comparison can be made to Attachment Based Family Therapy (ABFT), which is a similarly manualized and evidenced based approach, which has shown impressive results with depressed and suicidal adolescents (20). Very briefly the main theoretical differences between BEST and ABFT would appear to be the former’s emphasis on discourse and narrative as the aspects it draws from attachment theory. However, both approaches have similar overall goals and what appear to be some similar techniques to reduce family conflict, promote affect regulation, build attachment relationships, and encourage adolescent autonomy on the basis of strengthened family relationships. A detailed comparison of the two approaches would be a promising avenue for future research.

Treatments for adolescent substance use will clearly benefit from strategies designed to enhance not only attachment security, but the organisation of attachment related discourse. Such changes provide the secure family-base which enables an adolescent’s continuation of the developmental process into adulthood. Underlying attachment vulnerabilities are maintained not only in the representational models of the individual members, but also as interactional patterns and modes of communication within families. We propose that this discursive level of the family system can be a target of a number of specific techniques. Families as a whole can be engaged in these techniques and new approaches to patterns of communication and connectedness used as a means of engaging substance using, depressed, or suicidal adolescents.

## Author Contributions

The author confirms being the sole contributor of this work and has approved it for publication.

## Funding

Evaluation studies of BEST have been funded by the Australian Research Council (grant number LP110200167) and also Beyondblue, Drummond St Services, Australian Drug Foundation, Turning Point and WA Department of Justice and supported by Deakin and Murdoch Universities.

## Conflict of Interest

The author declares that the research was conducted in the absence of any commercial or financial relationships that could be construed as a potential conflict of interest.
